# What worsens swallowing in esophageal achalasia? Insights from patient-reported outcomes

**DOI:** 10.3389/fnut.2026.1706422

**Published:** 2026-01-30

**Authors:** Alessandra Cesarini, Giulia Scalese, Chiara Mocci, Lucia D’Alba, Carola Severi, Danilo Badiali, Emanuela Ribichini

**Affiliations:** 1 Department of Translational and Precision Medicine, Sapienza University, Rome, Italy; 2 Department of Gastroenterology and Digestive Endoscopy, Azienda Ospedaliera San Giovanni Addolorata, Rome, Italy

**Keywords:** dietary habits, dysphagia, esophageal achalasia, food rheology, nutritional assessment

## Abstract

**Introduction and aims:**

Esophageal achalasia (EA) is a rare motility disorder. Symptoms often impair quality of life (QoL) and lead to restrictive, self-managed diets with potential nutritional deficiencies. The study aimed to assess dietary patterns and nutritional status in EA patients.

**Materials and methods:**

EA patients, retrospectively recruited from January 2018 to August 2024, filled out a 15-day diary to record ingested food and relative symptoms onset for each meal. Estimated caloric intake and macronutrient composition were compared to those recommended by the Italian Society of Human Nutrition (SINU). EA activity was assessed with Eckardt Symptoms Score (ESS) and QoL with the MD Anderson Dysphagia Inventory (MDADI).

**Results:**

Of 44 patients (24M, 20F; 56.9 ± 15.7 years), 79% had active disease (ESS ≥3). The mean daily caloric intake was 1,573 ± 368 kcal/die, significantly lower than the estimated needs (*p* < 0.0001). Macronutrients distribution was unbalanced with an increase in fats (37.8%), a decrease in carbohydrates (43.2%), and insufficient fiber intake (14 g). The most common symptom-triggering foods were bread, pasta, pizza (50–60%). Additionally, 60% reported worsened symptoms with cold foods, while 53% found relief with hot foods.

**Conclusion:**

This study highlights the pivotal role of dietary factors, particularly food consistency and temperature, in the management of EA, supporting the incorporation of individualized dietary counseling into standard EA care.

## 1 Introduction

Esophageal achalasia (EA) is a rare motility disorder characterized by the absence of peristalsis and impaired relaxation of the lower esophageal sphincter (LES), leading to dysphagia, regurgitation, and chest pain (1). The estimated annual incidence is 2 per 100,000 individuals, with a prevalence of 10 per 100,000 (2). Although the underlying pathophysiology involves the progressive degeneration of inhibitory neurons within the myenteric plexus, the role of food consistency and temperature in modulating symptom severity remains insufficiently investigated (3).

Food rheology significantly influences symptom onset and severity. Solid foods, due to their higher viscosity and greater esophageal transit resistance, are more likely than liquids to provoke dysphagia, chest pain, and regurgitation (4, 5) in esophageal motor dysfunction. In addition to texture, bolus temperature has been shown to influence esophageal motility (6, 7).

Given the strong association between dietary patterns and symptom expression, malnutrition remains a common and often underrecognized complication of EA. (8) Therefore, comprehensive nutritional assessment and individualized dietary modifications are essential components of symptom management and play a critical role in optimizing clinical outcomes for patients with EA.

## 2 Aims and methods

### 2.1 Study design and objectives

This observational study aimed to explore the dietary habits, nutritional intake, and deficiencies in patients with EA while investigating the relationship between food texture and temperature and the onset of symptoms such as dysphagia, regurgitation, and retrosternal pain. Additionally, the study sought to assess the impact of achalasia on quality of life (QoL) and its correlation with symptom severity and nutritional status. Patients were instructed to complete a 15-day food and symptom diary as an integral part of their clinical visit at our gastroenterology department. Diaries were initiated at the time of consultation and returned upon completion. Although the overall study design is retrospective observational, the food diary component was collected prospectively within routine clinical practice and subsequently analyzed retrospectively.

### 2.2 Patient selection

**Figure 1:**
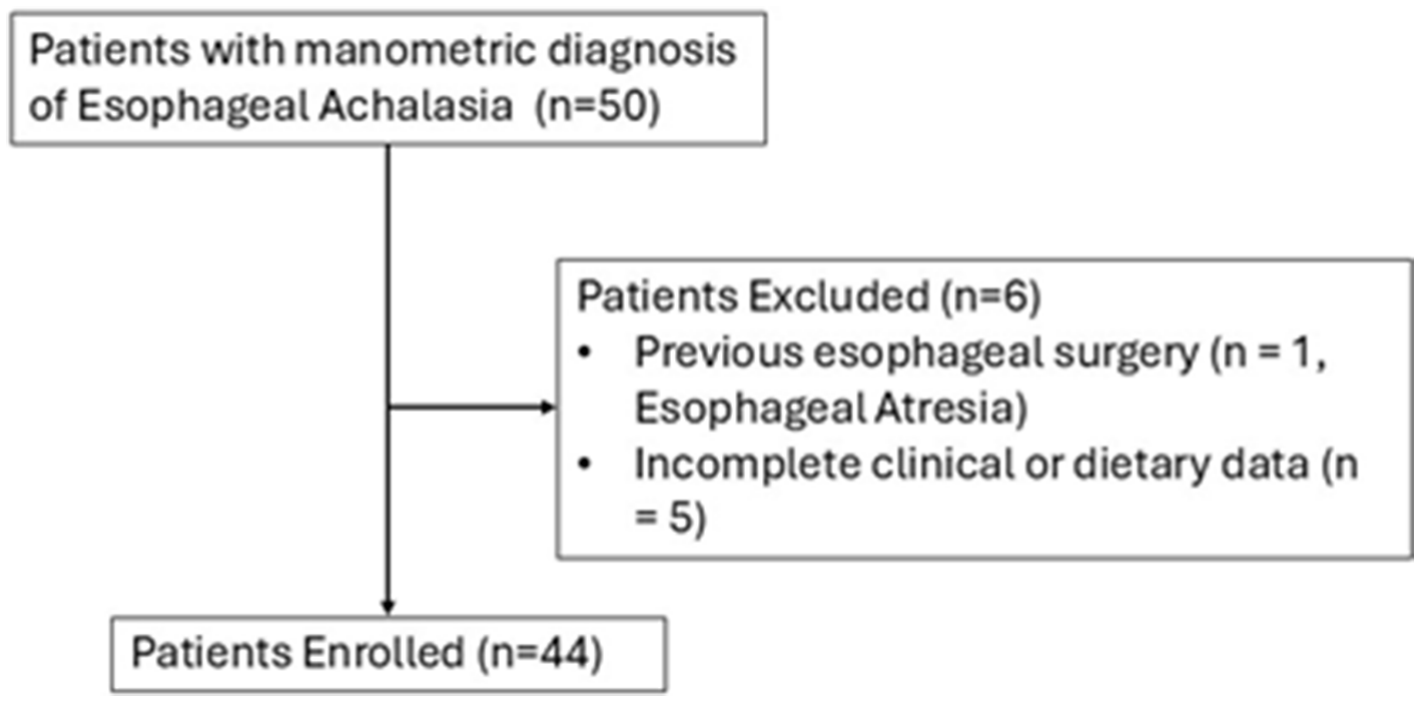
Study flowchart and patients’ selection.

Patients with a manometric diagnosis of EA were retrospectively selected from the Gastrointestinal Pathophysiology outpatient clinic at Policlinico Umberto I, Rome (30 January 2018–27 August 2024). The study included both newly diagnosed individuals and patients in follow-up after treatment. The diagnosis was made using esophageal high-resolution manometry (HRM) based on the Chicago Classification version 3.0 or 4.0 (9, 10), depending on the time of evaluation. Exclusion criteria included the presence of severe comorbidities, esophageal malignancy, previous esophageal surgery unrelated to EA, and incomplete clinical and dietary data. Detailed diagram illustrating patient selection is provided in Figure 1.

### 2.3 Treatment history and timing of evaluation

For each patient, the treatment history, including medical therapy, pneumatic dilation, surgical myotomy, peroral endoscopic myotomy (POEM), was recorded and compared to the date of questionnaire/diary completion. The time interval (months) between the last treatment and the clinical-nutritional evaluation was subsequently calculated.

### 2.4 Symptom and quality of life assessment

Symptom severity was assessed using the validated Eckardt Symptom Score (ESS), which quantifies symptom burden based on the presence and intensity of dysphagia, regurgitation, retrosternal pain, and weight loss. A total score of ≥3 was considered indicative of active disease (11). Quality of life was evaluated using the MD Anderson Dysphagia Inventory (MDADI), a symptom-specific questionnaire designed to measure the physical, emotional, and functional consequences of dysphagia. Based on their MDADI scores, patients were classified as having severe, moderate, mild, or no perceived disability (12). Detailed scores and corresponding levels of perceived disability are provided in Supplementary Table S1.

### 2.5 Dietary assessment

A 15-day food diary was used to evaluate habitual dietary intake and to identify symptom associations with specific food characteristics.

The food diary tracked dietary intake for three meals per day, resulting in a total of 45 meals recorded throughout the evaluation period. Patients were prospectively instructed to report all foods and beverages consumed, specifying portion sizes, preparation methods, and any associated symptoms such as dysphagia, regurgitation, and chest pain experienced during or after eating. Caloric intake and macronutrient composition, including carbohydrates, proteins, fats, and fiber, were assessed and compared to the Mediterranean diet recommendations (13) and the italian dietary reference intakes provided by the Italian Society of Human Nutrition (LARN) (14). Estimated energy requirements were calculated using the Harris and Benedict equation (15), adjusted for individual age, sex, and lifestyle activity factor. Special attention was given to food rheology, with consumed foods categorized as solid, semi-liquid, or liquid and to food temperature.

### 2.6 Statistical analysis

Data were analyzed with GraphPad Prism^©^ v9 using descriptive statistics to summarize clinical and dietary characteristics. Correlations between ESS and nutritional parameters (such as BMI, caloric intake, and macronutrient composition), and time passed since previous treatment were assessed using Pearson’s and Spearman correlation. Additionally, the relationships between food consistency, temperature, and symptom severity were examined through chi-square and Fisher’s tests for categorical data and Student’s *t*-tests or Mann–Whitney test for continuous variables, with a *p*-value <0.05 considered statistically significant. An analysis of variance (ANOVA) was performed when appropriate.

The study was approved by the formal Ethical Committee of Lazio (Italy) Sector 1, (Code: 7836) and conducted according to the ethical guidelines of the 1975 Declaration of Helsinki (6th revision, 2008). All participants provided written informed consent before inclusion.

## 3 Results

A total of 44 patients with EA (24M and 20F, mean age of 56.9 ± 15.7 years) were included. Concerning medical history, at the time of observation, 17 patients (39%) were newly diagnosed and had not yet undergone any treatment for EA, while the remaining 27 (61%) were follow-up patients who received prior treatment, either surgical, endoscopic or multiple treatment; in this subset of patients, the timing of diary completion varied by intervention type, with detailed intervals reported in Supplementary Table S2.

Regarding disease severity assessment, 35 patients (79%, 20 treated, 15 untreated) presented active disease (ESS ≥3), while the remaining 9 (21%, 7 treated, 2 untreated) had an ESS <3. The mean MDADI quality-of-life score was 19.95 ± 13.44. According to MDADI cut-offs (Supplementary Table S1), the mean score corresponds to a moderate level of perceived disability.

Untreated patients have significantly higher ESS and MDADI scores compared to treated patients (5.06 ± 2.4 vs. 3.56 ± 1.6 and 25.47 ± 15.2 vs. 16.6 ± 10.6, respectively) (*p* = 0.048 and *p* = 0.03).

Among treated patients, there was no significant correlation between the time elapsed since treatment and ESS values (*r* = 0.18, *p* = 0.36).

**Figure 2:**
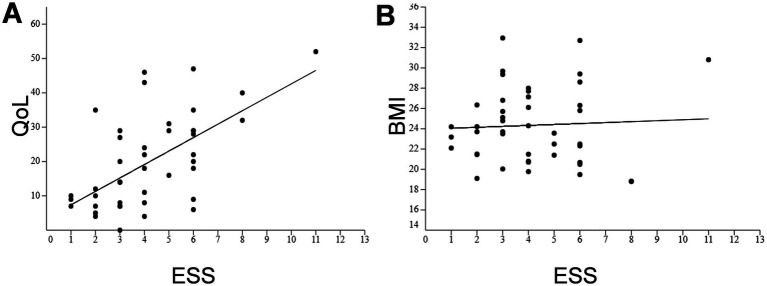
Correlation between symptom severity, quality of life (panel **A**), and body mass index (panel **B**) in patients with achalasia. Scatter plots showing correlations between clinical parameters in achalasia patients. Each dot represents one patient (*n* = 44). Pearson’s correlation (*r*) is reported for each comparison.

A positive correlation was found between ESS and perceived disability (*r* = 0.60, *p* < 0.0001), and no correlation was found between ESS and BMI (*p* = 0.75) (Figure 2).

### 3.1 Effect of food rheology on swallowing and related symptoms

Solid food was the prevalent symptom trigger, being associated with dysphagia, with an average of 16/45 meals (35.5%), chest pain (5/45 meals, 10%), regurgitation (4/45 meals, 8.9%) e cough (2/45 meals 5%). The association with other consistency is reported in Table 1.

**Table 1:** Frequency of symptom occurrence by food consistency.

Food consistency	Solid (%)	Semiliquid (%)	Liquid (%)	*p*-value
Dysphagia (%)	35.5	3.5	5.7	*p* < 0.001
Chest pain (%)	10	1.1	0.2	*p* < 0.001
Regurgitation (%)	8.9	1.4	1.9	*p* < 0.001
Cough (%)	5	0.2	0.6	*p* < 0.001

Percentages indicate the average proportion of 45 meals in which each symptom was reported, according to food texture.

**Figure 3:**
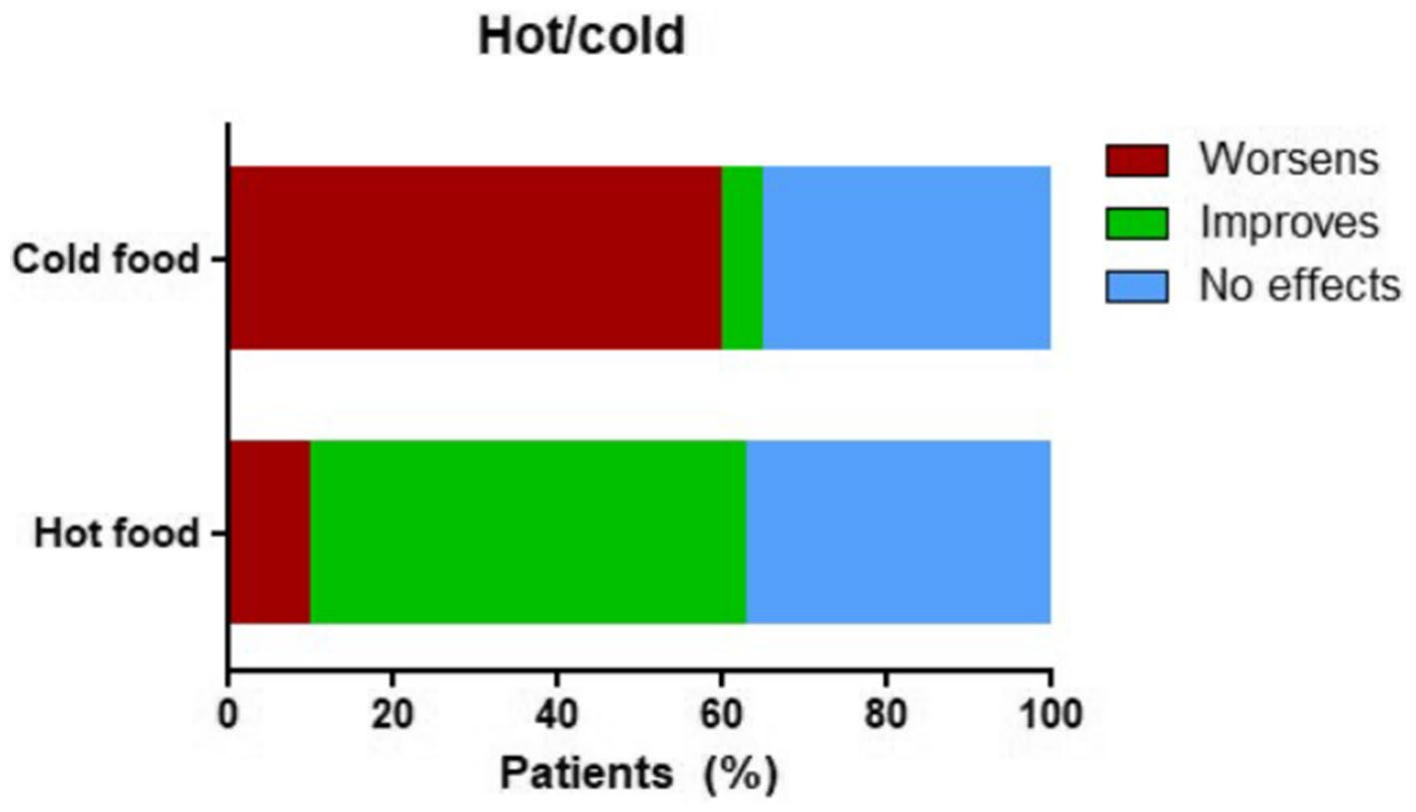
Perceived impact of food temperature on swallowing symptoms in achalasia patients. Stacked horizontal bar chart showing the percentage of patients reporting changes in swallowing symptoms after ingesting cold (top bar) or warm (bottom bar) foods and beverages.

Thermal properties of food and beverages also influenced symptom perception. Cold items were reported to worsen swallowing in 27 patients (60%), while 15 patients (35%) reported no change and 2 patients (5%) experienced improvement (*p* < 0.001). Conversely, warm foods and beverages were associated with symptom improvement in 24 patients (55%), no change in 16 patients (30%), and symptom worsening in 4 patients (15%) (*p* < 0.001) (Figure 3).

Notably, no statistically significant differences were observed in the effect of temperature on symptoms when stratifying patients by ESS (Supplementary Table S3).

### 3.2 Energy and macronutrient intake

The analysis of dietary diaries revealed a significant caloric deficit, with mean energy intake falling well below estimated requirements in both males and females (average deficit approximately 710 kcal/die, *p* < 0.0001). Macronutrient distribution showed an excess in fat intake, reduced consumption of carbohydrates and dietary fiber, and a slightly elevated protein intake relative to dietary recommendations. No significant differences were observed between males and females in caloric and macronutrient composition.

When stratifying patients by disease activity, patients with active disease had a greater caloric deficit and lower protein intake than patients with inactive disease, but without reaching statistical significance, while fiber intake was significantly lower (Table 2).

**Table 2:** Caloric intake and macronutrient composition compared to estimated needs: overall description and differences between patients with active or inactive disease according to ESS.

Parameter	Overall	Active disease (ESS ≥3; *n* = 35)	Inactive disease (ESS <3; *n* = 9)	Reference values (13, 14)	*p*-value
Mean caloric intake (kcal)	1,573 ± 368	1,545 ± 380	1,683 ± 316	Varies by age, sex and physical activity	0.27
Estimated caloric needs (kcal)	2,287 ± 373	2,287 ± 397	2,281 ± 273	Calculated individually	NA
Caloric deficit (kcal)	713 ± 415	742 ± 418	598 ± 404	NA	0.24
Carbohydrates (%)	43.2 ± 7.2	43 ± 7.5	44.2 ± 6.3	45–60%	0.61
Protein (%)	18 ± 4.5	17.3 ± 3.9	20.7 ± 6	15–20%	0.06
Total fat (%)	37.8 ± 7.7	38.6 ± 7.9	34.6 ± 6.5	25–35%	0.20
Fiber (g)	14 ± 5.2	**12.8 ± 5.1**	**18.3 ± 2.5**	25–30 g/day	**0.003**

The bold value refers to the *p*-value of fiber intake and highlights the only statistically significant difference between active and inactive disease groups.

### 3.3 Causes of incomplete meals in achalasia

Difficulty in completing meals was reported by 11 patients (25%). Among these, vomiting was cited as the main limiting factor in 5 patients (45%), followed by chest pain in 4 (36%) and regurgitation in 2 (18%).

Pain during ingestion was reported by 13 patients (30%) at least once daily, with persistent pain (lasting ≥6 months) present in 11 of them (86%). Chest pain was noted in 28 patients (64%), typically occurring several hours post-meal in 10 (36%), or during the night in 13 (46%), while only 5 (18%) experienced pain during or immediately after eating (*p* < 0.001).

Regurgitation affected 30 patients (68%), most commonly immediately after meals in 13 (43%) or at night in 10 (33%). Most of them (21 patients, 70%) could accurately describe its characteristics, reporting viscous material in 10 (48%), food residues in 9 (43%), and acidic content in 2 (9%).

A globus sensation was experienced by 18 patients (41%) more than once weekly and reported as a persistent symptom by 15 of them (83%) lasting ≥6 months. This symptom predominantly occurred between meals in 8 patients (44%), suggesting ongoing esophageal stasis rather than acute post-swallow onset.

**Figure 4:**
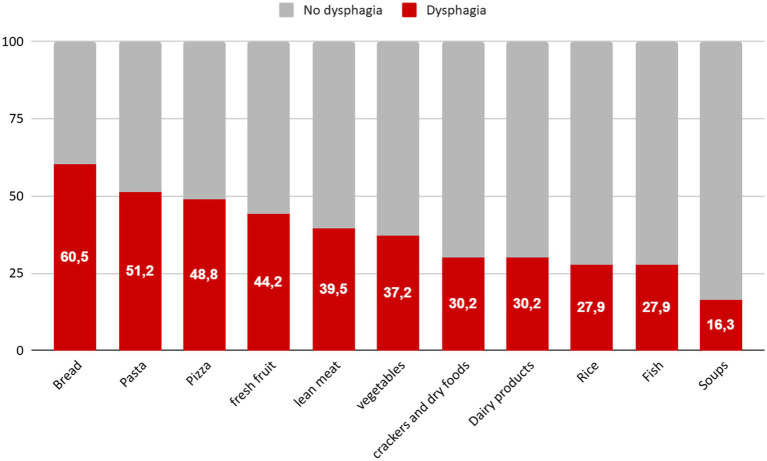
Food items associated with dysphagia. Percentage of patients who reported at least one episode of dysphagia after ingestion of each food item.

Participants reported that soft, moist foods, such as cooked vegetables, porridge, soups, and milk-based items, were most effective in facilitating swallowing. In contrast, hard or dry-textured foods (e.g., crackers, hard fruits, bread, and meat), as well as certain beverages like carbonated drinks, were more frequently associated with dysphagia (Figure 4).

## 4 Discussion

Esophageal achalasia is defined by the absence of peristalsis and incomplete relaxation of LES, leading to impaired bolus transit and symptomatology dominated by dysphagia (1). While a range of endoscopic and surgical treatments are available, dysphagia often persists or recurses, highlighting the need for adjunctive strategies to improve symptom control and nutritional status (8).

Despite the recognized impact of esophageal motility disorders on diet, no standardized nutritional guidelines currently exist for managing dysphagia in EA. Dietary recommendations are typically empirical, based on presumed pathophysiological mechanisms rather than evidence-based strategies. It is also worth noting that much of the dietary guidance available online or in clinical resources regarding dysphagia primarily refers to oropharyngeal or neurologically mediated dysphagia, where issues like dual-consistency textures and aspiration risk are predominant. In contrast, esophageal dysphagia, particularly that resulting from motility disorders such as EA, presents with distinct pathophysiological mechanisms and symptom triggers, primarily related to impaired bolus transit and esophageal stasis rather than coordination of swallowing phases. Therefore, general recommendations such as avoiding mixed consistencies (e.g., liquids with solids) or relying solely on thickened fluids may not be applicable and could be unnecessarily restrictive for this population.

To our knowledge, this is the first study to systematically observe real-life dietary patterns in EA patients and correlate the rheological properties of ingested foods with symptom expression, alongside detailed nutritional profiling while considering possible spontaneous adjustments adopted to manage the symptoms.

This study included a heterogeneous cohort of patients with EA at different stages of disease progression, encompassing both newly diagnosed individuals and those who had previously undergone various treatments (surgical or endoscopic), with variable intervals between intervention and evaluation. This variability reflects real-world clinical practice.

Notably, a substantial proportion of treated patients underwent only a single intervention (e.g., single pneumatic dilatation or partial myotomy), which may result in suboptimal symptom control. For patients with long intervals since treatment (>10 years), it is possible that disease progression, functional deterioration, or lack of dietary follow-up contributed to persistent symptoms. Although numbers in this subgroup are small, patients requiring multiple or early repeated interventions may represent a phenotype with more severe, refractory disease. We cannot exclude the possibility that very long intervals since the last treatment may influence symptom persistence, although the data are limited.

Furthermore, among treated patients, no significant correlation was found between the time elapsed since treatment and ESS scores indicating that time from treatment alone does not explain differences in symptom severity.

In addition to functional factors, persistent symptom activity in treated patients may also reflect structural remodeling of the esophageal wall. Histopathological studies have demonstrated smooth muscle and endomysial fibrosis in EA, particularly in long-standing disease, which may contribute to reduced compliance and persistent dysphagia despite technically adequate interventions (16, 17).

Subsequently, patients were stratified based on symptom burden using the ESS, a validated tool for assessing disease activity and guiding treatment decisions (11).

This strategy was selected to allow a comprehensive evaluation of the relationship between symptom expression and dietary behavior in real-life settings, irrespective of prior treatment, with a specific focus on food rheology and nutritional adequacy. Furthermore, it is well established that current therapies for EA are not definitive; the underlying pathophysiology persists, and symptoms may recur intermittently over time. Therefore, classifying patients based on their current symptomatology provides a clinically meaningful framework for evaluating real-life dietary responses and symptom expression, regardless of prior treatment status.

Given all these considerations, symptom burden was considerable in this cohort: most patients exceeded the clinical threshold for active disease, with a strong correlation between symptom severity and quality-of-life impairment.

Meal interruption was frequently attributed to upper gastrointestinal symptoms, most notably vomiting, chest pain, and regurgitation. Pain and regurgitation were often delayed or nocturnal, likely reflecting food stasis and prolonged esophageal clearance. However, the distinction between regurgitation and vomiting was not always clear, as some patients described emetic events in the absence of nausea or retching. This suggests that what was reported as “vomiting” may often represent passive regurgitation due to esophageal stasis rather than true gastric emesis. Likewise, reports of a “bolus” or “lump in the throat” are highly subjective and may reflect differing perceptions of the same symptom. These observations highlight the potential for reporting bias and underscore the importance of clear patient education and standardized terminology to enhance the accuracy of symptom assessment.

Notably, this cohort did not report paradoxical dysphagia, difficulty swallowing liquids but not solids, which is often described in the early stages of EA. This symptom typically reflects selective impairment of inhibitory neural pathways affecting lower esophageal relaxation (18). In this study, patients were assessed predominantly during chronic phases of the disease, either post-diagnosis or post-treatment. As such, symptom expression was more closely related to bolus consistency and mechanical clearance, with solids more frequently triggering symptoms.

Collectively, these findings underscore the clinical relevance of dietary modifications, not only in food texture and composition but also in temperature, in mitigating symptoms and preventing malnutrition. Routine dietary counseling and objective nutritional monitoring should be integrated into EA management, complementing medical or surgical therapies to improve functional outcomes and patient-reported quality of life (19).

Food consistency emerged as key modulators of symptom burden in EA. Solid, dry-textured items, especially starch-based foods like bread and pasta, were the most frequent dysphagia triggers, whereas soft or liquid preparations were generally better tolerated. These findings match previous pathophysiological evidence showing that solid foods exacerbate esophageal retention (4, 5). The observed dietary patterns also mirror data from prior research indicating that texture-modified diets enhance energy and protein intake while reducing aspiration risk (20), although the referenced meta-analysis included heterogeneous dysphagia populations and was not specific to EA.

Notably, our study also confirms the influence of food temperature. Cold foods and beverages were associated with worsened dysphagia and chest discomfort, while warm preparations appeared to alleviate symptoms.

Interestingly our findings indicate that the effect of food temperature on symptoms is independent of disease activity, suggesting that temperature-related symptom modulation is more closely linked to the underlying pathophysiology of EA rather than to symptom severity. This effect is likely attributable to temperature-induced manometric changes, as demonstrated by Ren et al. (6), who showed that cold swallows increase LES pressure and prolong esophageal contractions, while warm swallows promote LES relaxation and shorten contractile duration.

However, unlike Ren et al. (6), who relied on instrument-based assessments, our study offers complementary real-world evidence based on direct patient-reported experiences recorded over time. Thus, we reinforce and expand previous pathophysiological insights with observational data from daily life, strengthening the rationale for dietary modulation in the symptomatic management of EA.

Interestingly, rice and fish were among the foods least frequently associated with dysphagia in our analysis; however, this finding may be misleading. In clinical practice, rice is often perceived as problematic by patients with EA, as its granular texture and dry consistency can increase the risk of bolus retention. The apparent lower symptom frequency associated with rice in our cohort may reflect a behavioral avoidance: patients, when allowed a free diet, tend to exclude rice preemptively, alongside other high-risk foods, as a protective strategy. Therefore, its lower frequency in symptom reports likely represents underconsumption rather than true tolerance, introducing a potential bias that underscores the need for structured dietary assessment in EA.

In terms of nutritional intake, participants consumed substantially fewer calories than estimated requirements, with a dietary pattern characterized by elevated fat and reduced carbohydrate and fiber content compared with reference values (13, 14).

This behavior corroborates with a rational hypothesis grounded in the pathophysiology of EA: in the setting of persistently elevated LES pressure and impaired esophageal emptying, a low-fiber diet, typically defined as ≤10 g/day, may facilitate bolus transit (21). Soluble fiber increases bolus viscosity, while insoluble fiber contributes to luminal bulk through water retention, both of which could hinder esophageal clearance in a functionally obstructed esophagus. Similarly, the reduction in carbohydrate intake may reflect an avoidance of complex, dry starches such as bread and pasta, which were among the foods most frequently associated with symptom exacerbation in this cohort.

## 5 Strengths and real-world relevance

Although the clinical features of EA are well described, real-life data on dietary behaviors, food texture, and temperature-related symptom modulation remain scarce. The originality of the present study lies in the integrated evaluation of patient-reported dietary habits, symptom burden, and quality of life, providing clinically applicable insights that extend beyond purely pathophysiological descriptions.

The heterogeneity of the study population reflects real-world EA cohorts. Patients were not stratified solely according to treatment history but were classified based on current symptom activity using the ESS. Thus, the primary objective of the study was not to evaluate the response to therapeutic interventions, but rather to provide a cross-sectional snapshot of dietary habits in patients with EA who were symptomatic or asymptomatic at the time of assessment, irrespective of the type of prior intervention received. This approach allowed dietary behaviors to be interpreted in relation to the patient’s contemporaneous symptom burden, which is more relevant to everyday clinical management and nutritional counseling.

## 6 Limitations and future directions

Several limitations of this study should be acknowledged. The modest sample size, the retrospective design, and the reliance on self-reported dietary diaries represent inherent constraints. In addition, the timing of diary entries in relation to meals was not standardized, potentially introducing recall variability. The heterogeneity of the cohort, including differences in prior therapeutic interventions (surgical or endoscopic) and variable timing of diary completion relative to treatment, also limits direct comparability across patients.

Moreover, participants followed an unrestricted, self-selected diet, which may have led to unconscious avoidance of known symptom triggers and underreporting of problematic foods. While this reflects real-life adaptive eating behaviors, the lack of a fully standardized dietary questionnaire limits the uniform assessment of dietary habits and symptom associations.

Another limitation is that the potential influence of concomitant medications prescribed for comorbid conditions on swallowing function and esophageal symptoms was not systematically assessed. This may represent a source of residual confounding, as certain pharmacological agents could theoretically affect esophageal motility or sensory perception. However, the primary aim of the study was to capture patient-perceived associations between food characteristics and symptoms in a real-world setting, which may still reveal clinically meaningful patterns despite this variability.

Finally, symptom severity was assessed using the ESS. Recent psychometric analyzes have shown that the ESS has only fair reliability and construct validity, with dysphagia accounting for most of the explained variance, while chest pain and weight loss contribute marginally (11). Nevertheless, as dysphagia was the primary symptom of interest in the present study, the ESS remains a pragmatic and widely used clinical tool to stratify disease activity and to reflect symptom burden in routine practice.

Future research should include prospective, multicenter studies with larger cohorts, incorporating objective monitoring of standardized, prescribed diets and longitudinal assessment of dietary habits and symptom evolution before and after specific therapeutic interventions. Standardizing the timing of assessments relative to treatment would further improve the interpretation of post-intervention symptom changes and dietary behaviors.

## 7 Conclusion

This study highlights the pivotal role of dietary factors, particularly food consistency and temperature, in the management of EA. Our findings demonstrate that symptom burden is significantly modulated by dietary choices, and that many patients exhibit spontaneous adaptive behaviors that influence nutritional intake. These observations support the incorporation of individualized dietary counseling into standard EA care.

Furthermore, the strong correlation between symptom severity and impaired quality of life emphasizes the need for a multidisciplinary approach, integrating nutritional evaluation, psychological support, and clinical management to improve both functional outcomes and overall well-being.

## Data Availability

The raw data supporting the conclusions of this article will be made available by the authors upon reasonable request.
