# Supplementary material for: What worsens swallowing in esophageal achalasia? Insights from patient-reported outcomes
Source: Front Nutr. 2026 Jan 30;13:1706422. doi: 10.3389/fnut.2026.1706422 (PMC12900736; doi:10.3389/fnut.2026.1706422)
Supplement: Supplementary file 1 [file Table_1.doc]

**Supplementary Table S1. Corresponding scores for level of perceived disability
derived from the MD Anderson Dysphagia Inventory (MDADI) questionnaire.**

| **Score** | **Level of perceived disability** |
| --- | --- |
| 0-2 | Not perceived |
| 3-14 | Mild disability |
| 15-29 | Moderate disability |
| 30-57 | Severe disability |
| 58-60 | Total disability |

**Supplementary Table S2. Time interval between treatment and compilation of** the diet diary based on the type of treatment

| **Treatment** | **n** | **Time Interval (months)** | **Range  (month)** |
| --- | --- | --- | --- |
| **Pneumatic Dilatation (PD)** | 15 | 29 | 4-180 |
| **Lapoaroscopic Heller Myotomy (LHM)** | 8 | 15 | 5-228 |
| **PD+LHM** | 4 | 132 | 60-228 |

**Supplementary Table S3. Association between temperature and symptom occurrence according to disease activity.**

|  | **COLD FOOD** | | | **HOT FOOD** | | |
| --- | --- | --- | --- | --- | --- | --- |
|  | **ESS > 3** | **ESS < 3** | **p** | **ESS > 3** | **ESS < 3** | **p** |
| **Worsening** | **19** | **8** | **1.00** | **4** | **0** | **0.54** |
| **Improvement** | **2** | **0** |  | **18** | **6** |  |
|  | **21** | **8** | **29** | **22** | **6** | **28** |
